# Instability in the Penta-C and Penta-D Loci in Microsatellite-Unstable Endometrial Cancer

**DOI:** 10.3390/ijerph22111674

**Published:** 2025-11-04

**Authors:** Ahmet Yilmaz, Wendy L. Frankel, Weiqiang Zhao, Adrian A. Suarez, Wei Chen, Joshua F. Coleman, Joseph P. McElroy, Rachel Pearlman, Paul J. Goodfellow, Heather Hampel

**Affiliations:** 1 Department of Pathology, The Ohio State University Wexner Medical Center, Columbus, OH 43210, USAwendy.frankel@osumc.edu (W.L.F.); weiqiang.zhao@osumc.edu (W.Z.);; 2 Department of Pathology, University of Utah, Salt Lake City, UT 84112, USA; joshua.coleman@hsc.utah.edu; 3 Center for Biostatistics, Department of Biomedical Informatics, The Ohio State University College of Medicine, Columbus, OH 43210, USA; 4 Department of Internal Medicine, The Ohio State University Comprehensive Cancer Center, Columbus, OH 43210, USA; rachel.pearlman@osumc.edu; 5 Department of Obstetrics and Gynecology, The Ohio State University Wexner Medical Center, Columbus, OH 43210, USA; 6 Division of Clinical Cancer Genomics, Department of Medical Oncology and Therapeutics Research, City of Hope National Medical Center, Duarte, CA 91010, USA

**Keywords:** endometrial cancer, microsatellite instability, Penta-C, Penta-D, immunohistochemistry, DNA mismatch repair

## Abstract

Endometrial cancer (EC) is the most common gynecologic cancer. Early detection is one of the most important predictors of survival. The cancer is curable if detected early but the five-year survival rate in advanced cases can be as low as 22%. Microsatellite instability (MSI) testing is used to screen populations for Lynch Syndrome (LS), the most common cause of inherited EC, and to classify EC into distinct groups with unique histological, prognostic, and molecular features. Accurate sample identification is crucial for successful MSI testing because instability is assessed by comparing amplification patterns in markers in the normal and tumor samples that must be taken from the same individual. Penta-C and Penta-D pentanucleotide markers are used widely for sample identification in not only MSI testing but also parentage verification, forensic science, and population genetics studies. The objective of this study was to test 324 pairs of tumor and matched normal DNAs from EC patients for instability in these markers using the Promega MSI Analysis System^TM^ considered the “gold standard” in MSI testing. Both markers were unstable, and therefore not reliable for MSI testing, in 8.2% of the EC patients with MSI. Instability in both mono- and pentanucleotide markers suggest that the tumors with MSI likely suffer from a “generalized” form of instability also affecting other short tandem repeats. Results from many studies using these markers for various purposes may not be accurate if samples with MSI are involved.

## 1. Introduction

Microsatellites are short tandem repeats of nucleotides found throughout the genome [1]. Their repeat feature results in a “slippery” surface for DNA polymerase [2]. Replication errors due to DNA polymerase slippage resulting in deletions or duplications are common in microsatellites but the errors are precisely repaired by the mismatch repair (MMR) proteins mostly involving MLH1/PMS2 and MSH2/MSH6 heterodimers [3]. In MMR-deficient (dMMR) tumors, however, the replication errors remain unrepaired due to the missing or nonfunctional MMR proteins, resulting in microsatellite instability (MSI, non-matching microsatellite repeat size in the tumor and normal tissue or blood DNA taken from the same individual) [3]. The absence of functional MMR proteins may result from germline or somatic mutations in the MMR genes or promoter hypermethylation associated with transcriptional silencing of *MLH1*.

Endometrial cancer (EC) is the most common gynecologic cancer [4]. Early identification is one of the most important predictors of survival. The disease is curable through hysterectomy [5] if diagnosed early but the five-year survival rate in advanced cases is as low as 22% (https://www.cancer.org, accessed on 27 October 2025). MSI testing is used to screen populations for Lynch syndrome (LS), the most common cause of inherited EC, because approximately 90% of tumors in patients with LS are microsatellite-unstable [6]. Lynch syndrome is an autosomal-dominant disorder resulting from constitutive epimutations, germline mutations in one or more of *MLH1*, *MSH2*, *MSH6*, or *PMS2*, or abnormalities in *EPCAM* or other genes that regulate expression of MMR proteins [7].

MSI testing is additionally used to classify EC into distinct groups with unique histological, prognostic, and molecular features. EC was historically classified as type 1 (estrogen-dependent, low grade, seen in obese women, favorable prognosis) and type 2 (estrogen-independent, seen mostly in post-menopausal women, poor prognosis) [7]. However, this classification was inadequate because many high-grade cancers either overlapped both or did not fit into either category [7]. Currently, ECs have been categorized into tumors with MSI, POLE-mutated, and copy number-low, and -high groups [7,8]. More recently, in addition to The Cancer Genome Atlas (TCGA)-based classification of endometrial cancer [9] and the ProMisE and TransPORTEC systems, new approaches have emerged, including a one-step NGS-based method that classifies EC into molecular subtypes and evaluates their prognostic significance [10], as well as hierarchical foundation models leveraging whole-slide images [11]. However, these methods are not yet widely available in clinical practice. MSI testing is also used to identify patients who may be candidates for cancer immunotherapy. Due to the rich immune microenvironment in the tumor, EC patients with MSI tumors may benefit significantly from the anti-PD1 immunotherapy recently granted accelerated approval by the U.S. Food and Drug Administration [12]. PD-1 inhibitors, such as dostarlimab and pembrolizumab, show particularly strong therapeutic effectiveness in MSI-H or dMMR advanced endometrial cancers, with response rates ranging from 49% to 57%. PD-L1 inhibitors demonstrate lower effectiveness in MSS or PD-L1-positive advanced endometrial cancers, with response rates between 3% and 23% [13].

MSI testing methodologies have evolved over the years. An emerging method for diagnosing MSI is the Idylla MSI test, a fully automated assay that performs DNA extraction, amplification, and data analysis in approximately 150 min with minimal hands-on time and without the need for macro-dissection of tumor tissue. The system extracts DNA from formalin-fixed, paraffin-embedded (FFPE) tumor sections and amplifies seven monomorphic microsatellite biomarkers: ACVR2A, BTBD7, DIDO1, MRE11, RYR3, SEC31A, and SULF2. Fluorescent molecular beacons hybridize to the PCR products, and high-resolution melt curve analysis differentiates wild-type from the mutated sequences. Pattern recognition software interprets each biomarker’s mutation status, classifying samples as MSI-high (two or more mutated markers) or microsatellite stable (fewer than two mutations).

The Promega MSI Analysis System^TM^ is considered the “gold standard” in MSI testing [14]. The system includes five quasi-monomorphic mononucleotide repeat markers used for detection of MSI and two additional pentanucleotide repeat markers, Penta-C and Penta-D, used for sample identification. Accurate sample identification is crucial for successful MSI analysis because instability is assessed by comparing microsatellite repeat size in the normal and tumor samples that must be taken from the same patient. Penta-C and Penta-D are ideal for sample identification in this context because their repeat size is nearly identical in samples taken from the same individual but highly polymorphic in samples taken from different individuals. Penta-D is considered one of the “core loci” [15] commonly included in many commercial sample identification kits used for various purposes.

Despite their widespread use, we are not aware of any studies investigating genomic instability, which may result in sample mix-ups and invalidate the MSI test, in Penta-C and Penta-D in EC with MSI. Few colorectal cancer (CRC) studies have reported conflicting results [16,17]. The objective of this study was, therefore, to investigate the nature and frequency of instability in Penta-C and Penta-D in EC patients.

## 2. Materials and Methods

### 2.1. Patients, Sample Collection, and DNA Isolation

The 324 patients with valid results for Penta-C and Penta-D fragment size included in this study represent a subset of the EC patients described previously by Hampel et al. (2021) [18]. All of the patients included in this study were recruited at the Ohio State University Wexner Medical Center as described previously [18,19]. Institutional Review Board (IRB) approval for the OCCPI project was obtained from the Ohio State University (OSU) IRB (2012C0123). Written informed consent was obtained from all participants and/or their legal guardian(s). All methods were conducted in accordance with relevant guidelines and regulations. Surgically removed fresh normal and tumor tissue samples were formalin-fixed and paraffin-embedded using routine protocols. Pure sarcoma was excluded. Hematoxylin-eosin-stained slides were reviewed by pathologists and an area containing at least 30% viable tumor cells was marked for DNA isolation. On average, the samples contained 76.6% ± 15.5% viable tumor cells. All patients included in this study had normal tissue in addition to the tumor sample. QiaAmp DNA Micro (Qiagen, Germantown, MD, USA) or KingFisher Cell and Tissue DNA kit (Thermo Fisher, Waltham, MA, USA) were used to isolate DNA manually or using the KingFisher Flex DNA extractor (Thermo Fisher).

### 2.2. Microsatellite Instability Assay

A fluorescent multiplex PCR assay was carried out using the Promega MSI Analysis System^TM^ v1.2 (Madison, WI, USA) to amplify the mononucleotide repeat markers (with the gene overlapping the marker in parentheses) NR-21 (*SLC7A8*), BAT-26 (*MSH2*), BAT-25 (*c-kit*), NR-24 (*ZNF-2*), MONO-27 (*MAP4K3*), and the Penta-C (non-coding, AAAAG3–15) and Penta-D (non-coding, AAAAG2–17) pentanucleotide repeat markers. PCR products were detected by fluorescent fragment analysis (capillary electrophoresis) using the ABI 310 and 3130 genetic analyzers. The size distribution of the mono- and pentanucleotide markers were analyzed using GeneMapper v5 (Applied Biosystems, Carlsbad, CA, USA). The amplification patterns in mononucleotide repeat markers in the tumor were compared to the normal DNA reference and the samples were classified as MSI-H (MSI-high, two or more unstable markers), MSI-low (only one unstable marker), or MSS (microsatellite-stable, no unstable marker).

### 2.3. Classification of Peak Patterns in Mono- and Pentanucleotide Repeat Markers

**Figure 1:**
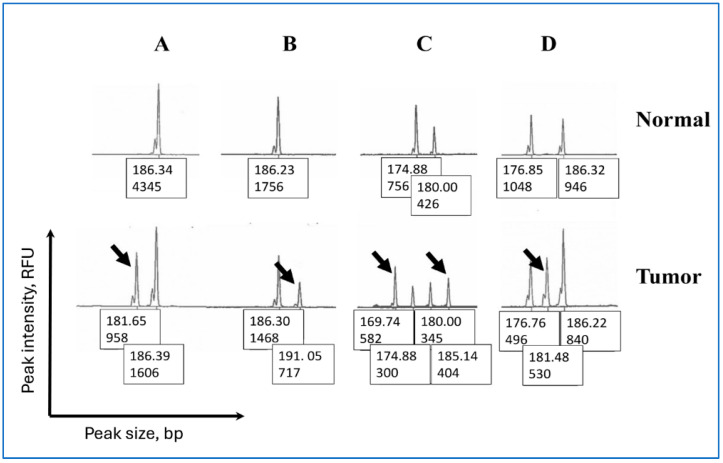
Representative examples of peak patterns in unstable Penta-C and Penta-D in endometrial cancer. The X- and Y-axes represent peak size in base pairs (bp) and peak height in RFU (Relative Fluorescent Units), respectively. The values displayed under each peak correspond to peak size and peak height. For example, in top row A, 186.34 denotes the peak size in bp, and 4345 denotes the peak intensity in RFU. The patterns include: (**A**) A deletion creating a new fragment approximately five bp shorter than the germline allele, (**B**) A duplication resulting in a new allele approximately five bp longer than the germline allele, (**C**) Different types of instability producing two new alleles. The 174 bp allele underwent deletion producing the 169 bp allele whereas the 180 bp allele underwent duplication producing the 185 bp allele. (**D**) A new 181 bp allele at equal distance to each of the two germline alleles is present. Samples with this type of peak pattern were included in the “Indeterminate” category because it is not possible to determine if the new allele is created as a result of a duplication in the 176 bp allele and/or a deletion in the 186 bp allele in the germline.

We are not aware of any previous studies classifying the distinct peak patterns observed in mono- and pentanucleotide repeat markers possibly because the previous studies had small sample sizes. Therefore, we classified the peak patterns in Penta-C and Penta-D into the following groups: “5 bp DEL” (a deletion in a sample with a single peak resulting in a new allele five bp shorter than the germline allele), “5 bp DUP” (a duplication in a sample with a single peak resulting in a new allele five bp taller than the germline allele), “5 bp DEL short” (a deletion in the shorter of two or more peaks resulting in a new allele five bp shorter than the germline allele), and “5 bp DUP tall” (a duplication in the taller of two or more peaks resulting in a new allele five bp taller than the germline allele). The samples were included in the “Indeterminate” category if a new allele appeared between germline alleles in a location that made it impossible to determine if the new allele was a result of deletion(s) and/or duplication(s) (Figure 1). For example, a new 170 bp allele appearing between 165 and 175 bp germline alleles was included in the “Indeterminate” category because it was not possible to determine if the 170 bp allele was the result of a duplication in the 165 bp allele and/or deletion in the 175 bp allele.

**Figure 2:**
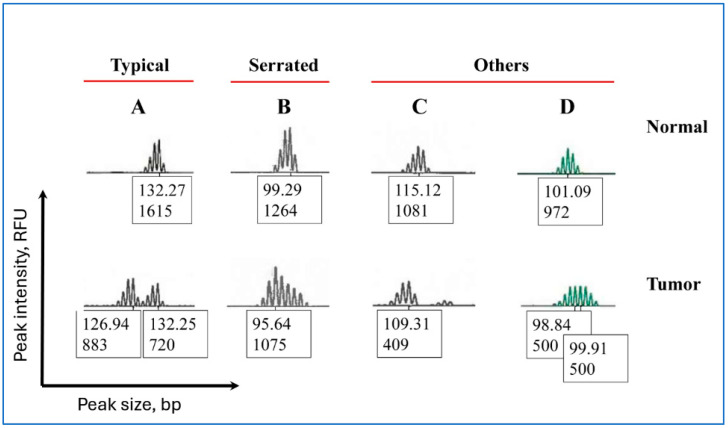
Representative examples of peak patterns in unstable mononucleotide repeat markers in endometrial cancer. The X- and Y-axes represent peak size in base pairs (bp) and peak height in RFU (Relative Fluorescent Units), respectively. The values displayed under each peak correspond to peak size and peak height. For example, in the top row A, 132.27 denotes the peak size in bp, and 1615 denotes the peak intensity in RFU. “Typical” (**A**) = two separate peaks with varying quantities of the normal- and tumor-specific fragments, “Serrated” (**B**) = peaks with incremental changes in peak heights and varying quantities of the normal- and tumor-specific fragments, “Others” = peaks that were neither “Typical” nor “Serrated”, including the peaks where a complete shift to the left on an electropherogram has occurred (**C**) due to the (nearly) total absence of the normal-specific peaks and those with fragments with similar heights located in the middle of the peak, resulting in a flat appearance (**D**).

Peak patterns in mononucleotide repeat markers were classified into the following groups (Figure 2): “Typical” (two separate peaks with varying quantities of the normal- and tumor-specific fragments), “Serrated” (peaks with incremental changes in fragment heights and varying quantities of the normal- and tumor-specific fragments), and “Others” (peaks that were neither “Typical” nor “Serrated”).

### 2.4. MLH1 Hypermethylation Assay

We used the Pyromark Q96 *MLH1* methylation assay (Qiagen) to test for hypermethylation at four CpG sites located at the *MLH1* promoter. Bisulfite conversion and amplification of the converted DNA and analysis of hypermethylation by pyrosequencing have been described previously [18,19]. In brief, 500 ng genomic DNA was converted using the EZ DNA Methylation Gold kit (Applied Biosystems). MLH1 sequences in bisulfite-treated DNA were amplified by PCR and the PCR products were run on a Pyromark Q96 ID pyrosequencer (Qiagen) following the manufacturer’s instructions with minor modifications. The methylation ratios at the four CpG sites were obtained by the Pyromark Q96 ID software v 2.5.10.7 (Qiagen). Fully methylated (SW48) and non-methylated (SW480) cell line DNA as well as 5% and 50% SW48 diluted in SW480 and no template controls were included in all assays.

### 2.5. Immunohistochemistry

Protocols used for the immunohistochemical staining of MLH1, PMS2, MSH2, and MSH6 and details of the antibodies used are available elsewhere [19]. The antibodies were purchased from Biocare Medical (Pacheco, CA, USA), Abcam (Cambridge, MA, USA), Vector Laboratories (Newark, CA, USA), or Cell Signaling Technologies (Danvers, MA, USA). In brief, the tissue sections cut in four-micron thickness were placed on positively charged glass slides. The slides were baked, cooled to room temperature, deparaffinized, dehydrated, and air-dried. After quenching, the slides were placed in an antigen retrieval solution, cooled, and processed using a Dako auto-stainer (Carpinteria, CA, USA).

### 2.6. Statistical Analysis

Fisher’s Exact or Chi-square test was used to analyze the variables using GraphPad Prism 5 (GraphPad, San Diego, CA, USA). When analyzing associations of mono- and pentanucleotide markers with clinical parameters including age at diagnosis, MLH1/PMS2 staining, *MLH1* hypermethylation status, etc. were considered a “family” and *P* values were adjusted (i.e., *P*_Adj_) to control the family-wise error rate using Holm’s procedure as described previously [20]. In brief, *P* values were sorted from smallest to largest.

$$P\left(1\right)\leq P\left(2\right)\leq \ldots \leq P\left(n\right)$$

where *n* represents the total number of comparisons with corresponding hypotheses *H*_(1)_, *H*_(2)_, …, *H*_(n)_. Each ordered *P* was compared to the threshold $\alpha^{\prime }$ defined as:

$$\alpha^{\prime }=\frac{\alpha }{k-1+1}$$

where *k* was the rank of the sorted *P* values (5 for the smallest, 4 for the second smallest, 3 for the third smallest *P*, etc. when five markers were included in the analysis). *H*_(i)_ was rejected if *P*_(i)_ was equal or less than the corresponding $\alpha^{\prime }$. The analysis was stopped at the first instance in which the null hypothesis (*H*_0_) was not rejected. The level of statistical significance was set at *P* < 0.05.

## 3. Results

### 3.1. Clinical and Pathological Characteristics of Patients

Overall clinical and pathological characteristics of patients included in this study are summarized in Table 1. Of all patients, 22.5% were MSI-H, 3.7% were MSI-low, and the remaining were MSS. Approximately 20.7% (67/324) of all patients and 79.8% (67/84) of those tested for hypermethylation were hypermethylated at the *MLH1* promoter. Of all patients, 21.9% were absent MLH1/PMS2 and 2.5% were absent MSH2/MSH6. Only PMS2 and only MSH6 were absent in 0.6% and 2.2% of the patients, respectively.

### 3.2. Both Penta-C and Penta-D Did Not Match in 8.2% of the MSI-H and 0.4% of the MSS Samples

Percent mismatches in pentanucleotide marker size between the normal and tumor tissue in the MSI-H, MSI-low, and MSS samples are presented in Table 2. When compared separately, Penta-C and Penta-D did not match in 16.4% and 26% of the MSI-H samples, respectively. These rates were 2.1% and 2.5% in MSS patients, respectively. When both Penta-C and Penta-D in the same patient were compared, mismatches were more frequent in MSI-H than the MSS samples (8.2% vs. 0.4%, *P* < 0.001), suggesting that the mismatches were likely due to genomic instability in MSI-H samples. When compared separately, Penta-C and Penta-D did not match in 33.3% and 25% of the MSI-low patients, respectively. All of the MSI-low patients matched when both Penta-C and Penta-D in the same patient were compared.

### 3.3. Peak Patterns in Unstable Penta-C and Penta-D Are Similar

Percent peak patterns in Penta-C and Penta-D did not differ significantly (*P* > 0.9, Table 3). The most frequent peak pattern (i.e., five bp deletion in the shorter of two or more peaks) was found in 28.6% of Penta-C and 17.9% of Penta-D reads. Percent viable tumor cells in samples with deletions, duplications, and “Indeterminate” patterns did not differ significantly (74.6%, 83.8%, and 82.5%, respectively, in Penta-C and 82.1%, 87.6%, and 74.3%, respectively, in Penta-D).

### 3.4. Penta-C Is More Stable than Penta-D

**Figure 3:**
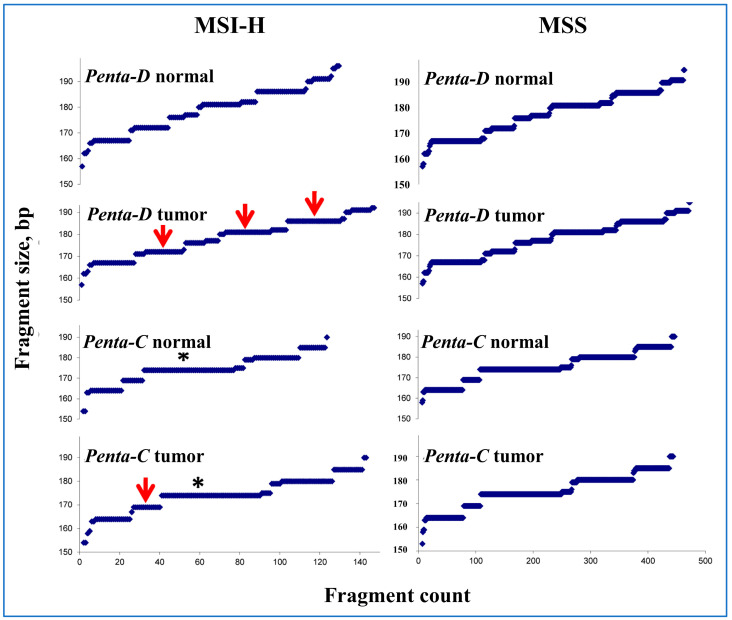
A visual representation of the PCR-amplified Penta-C and Penta-D fragments sorted by size. X- and Y-axes represent fragment count and fragment size in base pairs (bp), respectively. The arrows indicate gross dissimilarities between the normal and tumor samples in MSI-H samples. The asterisk denotes the 174 bp fragment found predominately in Penta-C but not in Penta-D. In MSI-H samples, the number of peaks in Penta-D in normal, Penta-D in tumor, Penta-C in normal, and Penta-C in tumor were 135, 158, 130, and 143, respectively. In MSS patients, these numbers were 446, 455, 439, and 444, respectively.

A plot of fragment size vs. count in Penta-C and Penta-D in normal and tumor tissue samples as a visual representation of all pentanucleotide fragments included in this study is presented in Figure 3. When the normal and tumor tissue in the MSI-H samples were grossly compared, an approximately 175 bp fragment in Penta-C and 170, 180, and 185 bp fragments in Penta-D were more abundant in tumor than the normal, suggesting that Penta-C is more stable than Penta-D. Half of the patients with mismatched Penta-C also had mismatched Penta-D but only 31.6% of the patients with mismatched Penta-D also had mismatched Penta-C, further suggesting that Penta-C is more stable than Penta-D in MSI-H samples.

The number of peaks in Penta-C and Penta-D fragments are presented in Supplementary Table S1. Consistent with the above observations, there were more triple peaks in Penta-D than Penta-C (20.5% vs. 11%) in MSI-H samples. Interestingly, none of the 324 normal tissue samples from the MSI-H or MSS patients had three or more peaks, suggesting that the presence of three or more peaks in normal tissue may be a marker for sample mix-ups and/or contamination in MSI testing in EC.

### 3.5. Clinical Characteristics in Patients with and Without Matching Penta-C and Penta-D

*MLH1* hypermethylation, and age at diagnosis were not significantly different in samples with and without mismatches in pentanucleotides (Table 4). However, there were more unstable mononucleotide markers in samples with than without mismatches in Penta-C (0.8 vs. 2.8) and Penta-D (0.7 vs. 2.9). In addition, the absence of MLH1/PMS2 staining was associated with increased mismatches in both pentanucleotides (*P* < 0.001), suggesting that the mismatches in Penta-C and Penta-D were likely due to dMMR. These differences were not related to the tumor content in the sample which ranged from 75% to 82% in all subgroups (Table 4).

### 3.6. Peak Patterns in Unstable BAT-25 Are Correlated with IHC Staining and MLH1 Hypermethylation

Associations of peak patterns in mononucleotide repeat markers with age at the time of diagnosis, IHC staining for MMR proteins, and *MLH1* hypermethylation are presented in Table 5. These associations were investigated because instability in Penta-C and Penta-D was associated with both IHC staining and increased instability in mononucleotide markers. Peak patterns were associated with MSH2/MSH6 staining and *MLH1* hypermethylation (*P*_Adj_ < 0.01); “Serrated” BAT-25 was found in all 19 samples with but none of those without MSH2/MSH6 staining. In addition, all but one of the 17 samples with “Serrated” peaks in BAT-25 were hypermethylated at the *MLH1* promoter (94.1% vs. 5.9%, *P*_Adj_ < 0.01), suggesting that *MLH1* hypermethylation and IHC staining were correlated, at least in part, with peak patterns in unstable mononucleotide repeat markers. Therefore, peak patterns in both mono- and pentanucleotide repeat markers are, at least in part, correlated with dMMR.

### 3.7. Sample Mix-Ups Due to Matching in Penta-C and Penta-D Size by Random Chance Are Negligible

The most common Penta-C and Penta-D combinations found in at least three patients included in this study are presented in Supplementary Table S2. Interestingly, a 174 bp fragment was present in 91.7% (22/24) of all allelic combinations in Penta-C but none in Penta-D. The most common combinations, found in five patients each, included the 164/174 bp and 174/180 bp fragment combinations in Penta-C and the 167/186 bp fragment combination and the 186 bp fragment in Penta-D.

## 4. Discussion

Representative examples of peak patterns in unstable mono- and pentanucleotide markers found in this study are presented in Figure 1 and Figure 2. Molecular events leading to the appearance of these peak patterns are poorly understood but it is assumed that the patterns are a product of the step-wise nature of MSI [21]. The changes in allele sizes are likely due to the sequential replication errors that progressively accumulate during multiple cell divisions [21]. The larger normal-specific alleles on the right side of an electropherogram are gradually replaced by the smaller tumor-specific alleles on the left, resulting in a shift from the right (larger germline allele) to the left (smaller tumor-specific peaks) on an electropherogram. It is assumed that the distinct peak patterns are created during this shift through unknown mechanisms.

In our study, Penta-C was more stable than Penta-D likely due to the presence of a 174 bp fragment found predominantly in Penta-C but not in Penta-D (Figure 3). Consistently, there are more triple peaks in Penta-D than Penta-C in MSI-H samples (11% vs. 20.5%, Supplementary Table S1), suggesting that increased variation in a pentanucleotide marker size may be associated with increased strand slippage during DNA replication. Additional potential biological explanations for the observed differences in stability between Penta-C and Penta-D include locus-specific sensitivity to MMR deficiency, effects of local sequence context on replication fidelity, allelic imbalance, epigenetic modifications, tumor heterogeneity, and technical factors related to PCR amplification and detection that may affect fragment size and counts. Interestingly, none of the 324 EC normal tissue samples contained three or more peaks in either of the pentanucleotide markers, suggesting that the presence of three or more peaks in the normal tissue in Penta-C or Penta-D may suggest contamination and/or sample mix-ups and invalidate the MSI test.

Penta-C and Penta-D size in the tumor and normal tissue samples did not match in 8.2% of the MSI-H samples (Table 2). Therefore, MSI analyses for 8.2% of the MSI-H samples would be subject to the concern of sample mix-ups if sample identification was solely based on marker size. We believe these mismatches in MSI-H samples are a manifestation of genomic instability in these tumors, rather than sample mix-ups and/or contamination, due to the following reasons: (1) all of the patients simultaneously underwent IHC staining and results concordant with MSI analysis were obtained, (2) each sample was labeled with detailed patient-identifying information such as name, date of birth, surgical pathology number, medical record number, etc., (3) PCR and DNA isolation were performed in different rooms, and all equipment was inspected and calibrated periodically, (4) although MSI-H, MSS, and MSI-low samples were processed simultaneously in random batches, pentanucleotide instability was significantly more frequent in MSI-H than the MSS samples, and (5) a subset of the MSI-H samples were submitted for ColoSeq testing at the University of Washington for verification. Our MSI testing and ColoSeq results were concordant in over 99% of the samples. In this study, we did not use blood DNA as the normal control because normal tissue was readily available for all patients. Using the blood DNA as normal control could unnecessarily increase the workload and sample mix-ups and/or contamination rates.

The absence of MLH1/PMS2 staining in IHC was associated with increased mismatches in Penta-C and Penta-D (*P* < 0.001), suggesting that pentanucleotide mismatches were associated with dMMR. IHC staining of the MMR proteins was also associated with peak patterns in mononucleotide repeat markers. “Serrated” BAT-25 peaks were present in all of the samples that were present but none of the samples that were absent MSH2/MSH6 staining and the peaks were found in all but one of the samples absent *MLH1* hypermethylation (94.1% vs. 5.9%, *P*_Adj_ < 0.01, Table 5). These results collectively suggest that peak patterns in both mono- and pentanucleotide markers are, at least in part, correlated with dMMR.

To our knowledge, this is the first study investigating the nature and extent of peak patterns in mono- or pentanucleotide repeat markers, or association of the peak patterns with clinical parameters in EC. Two previous colorectal cancer (CRC) studies investigating instability in Penta-C and Penta-D reported conflicting results. Murphy et al. (2006) [17] reported 36% and 45% allelic shifts in Penta-C and Penta-D, respectively, in MSI-H samples but the authors analyzed only 11 samples. An earlier study by Bacher et al. (2004) [16] reported lower rates of shifts (i.e., 14% and 35% in Penta-C and Penta-D, respectively) in 153 CRC tumors. The authors did not investigate frequency of the peak patterns or associations of the patterns with clinical parameters.

Our results suggest that repeat length, sequence context, and/or DNA repair deficiency may differentially affect pentanucleotide and mononucleotide markers. Mononucleotide markers are more likely to show instability and are more reliable than pentanucleotide markers for MSI detection. Mononucleotide repeats such as BAT25 and BAT26 tend to be especially sensitive to MMR deficiencies because the single-nucleotide repeat tracts are inherently less stable and more prone to errors that slip past defective repair enzymes, leading to frequent MSI in the absence of MMR proteins. Previous studies have shown that the short, contiguous runs of single bases in mononucleotide markers increase the risk of insertion–deletion mutations [22]. In contrast, pentanucleotide repeats, which are longer and more diverse, are likely more stable and less prone to replication errors than mononucleotide repeats. Slippage efficiency decreases with increasing repeat length and requires polymerase pausing and dissociation within the repeat tracts [23]. Pentanucleotide repeats, due to their longer repeat units, likely form slippage intermediates during replication that are either generated less frequently or recognized and corrected more efficiently by the replication machinery compared to mononucleotide repeats.

We believe the following results from our study will be especially helpful in the clinic: First, improvements are needed in MSI testing methodologies that involve Penta-C and Penta-D markers. As detailed above, MSI testing is used to screen populations for early identification of LS, determine the availability of treatment options, and classify the disease into distinct groups. Our results indicate that Penta-C and Penta-D in 8.2% of the MSI-H EC patients are not reliable for MSI testing. Results from many commercial sample identification kits based on these markers used widely for various purposes may not be accurate if samples with dMMR are involved.

Second, our results suggest that sample mix-ups in clinical laboratories are probably infrequent in samples without MSI. Both Penta-C and Penta-D did not match in only 0.4% of the 239 MSS samples; this rate is similar to the 0.5% sample mix-up rate reported in clinical laboratories and the 0.2 to 0.6% error rate reported by the manufacturer of the MSI Analysis System. Therefore, pentanucleotide markers are probably reliable for the identification of MSS samples, and the 0.4% mismatch rate we found likely represents either true sample mix-ups or the limit of sample identification based on these pentanucleotide repeat markers.

Detection of sample mix-ups and/or contamination in clinical laboratories has remained a persistent and challenging task rarely investigated and likely underreported [24]. However rare they may be, sample mix-ups may result in serious harm to the patient. We are aware of only few studies investigating sample mix-ups in clinical laboratories. The U.S. Government Accountability Office (https://www.gao.gov, accessed on 2 November 2025) has estimated that sample mix-ups occur in up to 2.3% of the samples in clinical laboratories. In 2018, 3.8% (364/9655) of all clinical laboratories inspected by the Centers for Medicare & Medicaid Services (CMS) were cited due to violating regulations related to specimen source errors. In clinical laboratories, sample mix-ups may occur in up to 3.5% of samples, and it is estimated that up to 1.3% of prostate cancer biopsies are false positives due to sample mix-ups and/or contamination [25]. Several STR panels have been developed specifically to detect sample mix-ups in pathology laboratories [26,27].

The third result from our study that may have clinical relevance is that the peak patterns in both mono- and pentanucleotide markers are correlated with IHC staining and *MLH1* promoter hypermethylation. *MLH1* hypermethylation is a common occurrence in MSI-H EC. In our study, 79.8% (67/84) of the patients tested were hypermethylated at the MLH1 promoter. This result is in line with Goodfellow et al. (2003; 72.4%, 92/127) [28], Buttin et al. (2004; 71.2%, 79/111) [29], Pauly et al. (2021; 81.4%, 22/27) [30], and Peterson et al. (2012; 82.8%, 24/29) [31] who reported similar *MLH1* hypermethylation rates in MSI-H EC. We are not aware of any studies investigating molecular mechanisms resulting in appearance of these peak patterns that were independent of the tumor cell content in our study.

Several limitations of research are evident in our study. Caution should be exercised when interpreting our results regarding peak patterns in mononucleotide repeats which may be subjective due to the small size of allelic shifts and potential “bleed-through” in some peaks. The sample size in some subcategories in the analysis of IHC results and patient race was small. There were no mismatches in any of the MSI-low samples when repeat size in both pentanucleotides were compared (Table 2). Although interesting, this result may be due to the small sample size in the MSI-low group (*N* = 73, 239, and 12 in MSI-H, MSS, and MSI-low patients, respectively). Additionally, our results have not yet been validated in independent endometrial cancer cohorts. Furthermore, because 96.6% of the patients in this study were White, the data do not allow for meaningful conclusions regarding the impact of race. It should be noted that MSI-low samples are rare in EC. Allelic imbalance, copy number variations, loss-of-heterozygosity, or other complex genetic alterations were not investigated in our study.

In conclusion, our results suggest that instability in pentanucleotide repeat markers in MSI-H tumors is associated with dMMR as it is correlated with both MSI and the absence of IHC staining for MMR proteins. Penta-C and Penta-D are not reliable for sample identification in 8.2% of EC patients with MSI. Results from many studies employing these pentanucleotides for sample identification should be interpreted with caution if samples with dMMR are involved; sample identification should be verified using alternative methods. To our knowledge, this is the first study investigating peak patterns in unstable mono- and pentanucleotide repeat markers used for MSI analysis in EC and may prove useful as a foundation for future studies with larger sample size.

Future research should investigate whether these observations occur in other tumor types and continue refining molecular diagnostic approaches to enhance sensitivity and accuracy in clinical applications. The broader integration of emerging technologies such as digital PCR and next-generation sequencing could enable higher-resolution detection of MSI patterns, particularly in cases with subtle microsatellite shifts that are currently unrecognized. Furthermore, large-scale, population-based studies are necessary to systematically define the clinical relevance of distinct MSI shift profiles and their associations with dMMR.

## Figures and Tables

**Table 1:** Clinical characteristics of the 324 endometrial cancer patients included in this study.

Parameter		*N*	%
Age younger than 50 years		43	13.3
MSI status			
	MSI-H	73	22.5
	MSI-low	12	3.7
	MSS	239	73.8
*MLH1* promoter hypermethylation			
	Present	67	20.7
	Absent	17	5.2
Immunohistochemistry			
	Absent MLH1/PMS2	71	21.9
	Absent MSH2/MSH6	8	2.5
	Absent PMS2 only	2	0.6
	Absent MSH6 only	7	2.2
	IHC intact	233	71.9
	Others	3	0.9
Race			
	White	313	96.6
	Black	7	2.2
	Asian	3	0.9
	Unknown	1	0.3

**Table 2:** Percent matching in Penta-C and Penta-D size between the normal and tumor in MSI-H, MSI-low, and MSS samples.

			Penta-C				Penta-D				Both Penta-C and Penta-D							
			Match		Does Not Match		Match		Does Not Match		Match		Do Not Match		Only Penta-C Does Not Match		Only Penta-D Does Not Match	
	*N*	TC	*N*	%	*N*	%	N	%	*N*	%	*N*	%	*N*	%	*N*	%	*N*	%
MSI-H	73	79.7	61	83.6	12	16.4	54	74	19	26	48	65.8	6	8.2	6	8.2	13	17.8
MSI-low	12	73.5	8	66.7	4	33.3	9	75	3	25	5	41.7	0	0.0	4	33.3	3	25.0
MSS	239	76.7	234	97.9	5	2.1	233	97.5	6	2.5	229	95.8	1	0.4	4	1.7	5	2.1

**Table 3:** Peak patterns in Penta-C and Penta-D in endometrial cancer samples grouped based on the presence of a single or two or more peaks in the normal tissue. TC = percent viable tumor cells.

		Penta-C			Penta-D		
Peak Patterns		TC	*N*	%	TC	*N*	%
Normal tissue has a single peak							
	5 bp deletion	68.3	3	14.3	85	3	10.7
	5 bp duplication	90	1	4.8	80	2	7.1
	10 bp deletion	Not available	1	4.8	80	1	3.6
	Indeterminate	Not available	0	0.0	70	1	3.6
Normal tissue has two or more peaks							
	5 bp deletion in the shorter fragment	76	6	28.6	82	5	17.9
	5 bp duplication in the longer fragment	81.7	3	14.3	90.8	4	14.3
	1 bp duplication in the shorter fragment	Not available	0	0	90	1	3.6
	5 bp deletion in the longer fragment	90	2	9.5	81	5	17.9
	Indeterminate	73.8	5	23.8	75	6	21.4
Total deletions		74.6	12	57.1	82.1	14	50
Total duplications		83.8	4	19.1	87.6	7	25
Total “Indeterminate”		82.5	5	23.8	74.3	7	25
Total		77.5	21	100	81.5	28	100

**Table 4:** Associations of instability in Penta-C and Penta-D with clinical parameters in endometrial cancer.

		Penta-C				Penta-D				Both Penta-C and Penta-D							
		Matches		Does Not Match		Matches		Does Not Match		Match		Do Not Match		Only Penta-C Does Not Match		Only Penta-D Does Not Match	
		*N*	%	*N*	%	*N*	%	*N*	%	*N*	%	*N*	%	*N*	%	*N*	%
TC%		77		78		77		82		77		82		75		81	
UNS		0.8		2.8		0.7		2.9		0.7		4.1		2.1		2.5	
HYP																	
	Present	55	82.1	12	17.9	49	73.1	18	26.9	41	61.2	4	6	8	11.9	14	20.9
	Absent	15	88.2	2	11.8	12	70.6	5	29.4	12	70.6	2	11.8	0		3	17.6
AAD																	
	<50 yr	41	95.3	2	4.7	41	95.3	2	4.7	40	93	1	2.3	1	2.3	1	2.3
	≥50 yr	261	93.2	19	6.8	254	90.7	26	9.3	241	86.1	6	2.1	13	4.6	20	7.1
Absent IHC																	
	MLH1/PMS2	59	83.1	12	16.9	53	74.6	18	25.4	45	63.4	4	5.6	8	11.3	14	19.7
	MSH2/MSH6	7	87.5	1	12.5	6	75	2	25	6	75	1	12.5	0	0	1	12.5
	PMS2 only	2	100	0	0	0	0	2	100	0	0	0	0	0	0	2	100
	MSH6 only	6	85.7	1	14.3	7	100	0	0	6	85.7	0	0	1	14.3	0	0
	Others	2	100	0	0	2	100	0	0	2	100	0	0	0	0	0	0
	IHC intact	226	97	7	3	227	97.4	6	2.6	222	95.3	2	0.9	5	2.1	4	1.7

Abbreviations used: TC = tumor content, %, UNS = average unstable mononucleotide repeat markers (out of 5), HYP = *MLH1* hypermethylation, AAD = age at diagnosis, <50 yr = Younger than 50 years at the time of diagnosis, ≥50 = fifty years or older at the time of diagnosis, IHC = Immunohistochemistry.

**Table 5:** Associations of peak patterns in mononucleotide repeat markers with clinical parameters in endometrial cancer.

			Age at Diagnosis				MLH1/PMS2 Staining				MSH2/MSH6 Staining				*MLH1* Hypermethylation			
			<50 yr		>50 yr		Present		Absent		Present		Absent		Present		Absent	
Pattern		TC	*N*	%	*N*	%	*N*	%	*N*	%	*N*	%	*N*	%	*N*	%	*N*	%
NR21																		
	Serrated	81.9	3	21.4	11	78.6	2	12.5	14	87.5	15	88.2	2	11.8	11	78.6	3	21.4
	Typical	76.3	0	0	12	100	2	18.2	9	81.8	9	75	3	25	8	66.7	4	33.3
	Other	80.75	2	10	18	90	1	5	19	95	20	100	0	0	17	85	3	15
NR24																		
	Serrated	79.9	2	8	23	92	4	16	21	84	22	91.7	2	8	17	68	8	32
	Typical	82.1	2	15.4	11	84.6	5	38.5	8	61.5	10	76.9	3	23.1	7	53.8	6	46.2
	Other	81.6	2	12.5	14	87.5	1	5.88	16	94.1	16	94.1	1	5.88	14	87.5	2	12.5
BAT25																		
	Serrated	75.3	0	0	17	100	2	10	18	90	19	100	0	0	16	94.1	1	5.9
	Typical	82.3	2	18.2	9	81.8	4	44.4	5	55.6	6	60	4	40	3	27.3	8	72.7
	Other	84.04	3	11.5	23	88.5	5	17.2	24	82.8	25	89.3	3	10.7	21	80.8	5	19.2
BAT26																		
	Serrated	70.6	2	14.3	12	85.7	4	23.5	13	76.5	15	100	0	0	11	78.6	3	21.4
	Typical	82.3	1	4.5	21	95.5	2	9.52	19	90.5	19	86.4	3	13.6	15	68.2	7	31.8
	Other	82.4	5	16.7	25	83.3	7	21.2	26	78.8	27	87.1	4	12.9	24	80	6	20
MONO27																		
	Serrated	78.8	3	15	17	85	7	30.4	16	69.6	18	85.7	3	14.3	13	65	7	35
	Typical	71.3	1	5.6	17	94.4	2	11.8	15	88.2	15	83.3	3	16.7	13	72.2	5	27.8
	Other	84.8	1	9.1	10	90.9	0	0	10	100	11	100	0	0	7	63.6	4	36.4

Pattern = peak patterns in mononucleotide repeat markers, TC = percent tumor content, <50 yr = age younger than 50 years at the time of diagnosis, >50 yr = age older than 50 years at the time of diagnosis, Serrated = serrated peaks with varying quantities of the normal- and tumor-specific fragments, Typical = separate peaks with varying quantities of the normal- and tumor-specific fragments, Others = peaks that were neither “Typical” nor “Serrated”, including the “Flat” peaks with fragments with similar heights located in the middle of the peak, resulting in a flat appearance and the peaks where a complete shift to the left on an electropherogram occurred due to the (nearly) total absence of the normal-specific peaks. Representative images of these peak patterns are presented in Figure 2.

## Data Availability

Data supporting the findings of this study are available from the corresponding author, Heather Hampel, upon reasonable request.

## Supplementary Materials

The following supporting information can be downloaded at: https://www.mdpi.com/article/10.3390/ijerph22111674/s1, Table S1: Number of peaks in Penta-C and Penta-D in MSI-H, MSI-low, and MSS patients with endometrial cancer; Table S2. Most common Penta-C and Penta-D combinations seen in at least three patients with endometrial cancer. *N* = patients with the same Penta-C/Penta-D combination.
