# Supplementary material for: Instability in the Penta-C and Penta-D Loci in Microsatellite-Unstable Endometrial Cancer
Source: Int J Environ Res Public Health. 2025 Nov 4;22(11):1674. doi: 10.3390/ijerph22111674 (PMC12652968; doi:10.3390/ijerph22111674)
Supplement: Supplementary file 1 [file ijerph-22-01674-s001.zip › ijerph-3854422-supplementary.pdf]

Supplementary Materials

Supplementary Table S1. Number of peaks in Penta-C and Penta-D in MSI-H, MSI-low, and MSS patients with endometrial cancer

			Single peak		Double peaks		Three or more peaks	
MSI status	Marker	Tissue type	*N*	%	*N*	%	*N*	%
MSI-H (*N* = 73)	Penta-D	Normal	15	20.5	58	79.5	0	0
		Tumor	10	13.7	48	65.8	15	20.5
	Penta-C	Normal	22	30.1	51	69.9	0	0
		Tumor	18	24.7	47	64.4	8	11
MSI-Low (*N* = 12)	Penta-D	Normal	2	16.7	10	83.3	0	0
		Tumor	2	16.7	7	58.3	3	25
	Penta-C	Normal	5	41.7	7	58.3	0	0
		Tumor	4	33.3	5	41.7	3	25
MSS (*N* = 239)	Penta-D	Normal	49	20.5	190	79.5	0	0
		Tumor	47	19.7	188	78.7	4	1.7
	Penta-C	Normal	52	21.8	187	78.2	0	0
		Tumor	54	22.6	182	76.2	3	1.3
All patients (*N* = 324)	Penta-D	Normal	66	20.4	258	79.6	0	0
		Tumor	59	18.2	243	75.0	22	6.8
	Penta-C	Normal	80	24.7	244	75.3	0	0
		Tumor	76	23.5	234	72.2	14	4.3

Supplementary Table S2. Most common Penta-C and Penta-D combinations seen in at least three patients with endometrial cancer. *N* = patients with the same Penta-C/Penta-D combination

N	Penta-C normal size, bp	Penta-C tumor size, bp	Penta-D normal size, bp	Penta-D tumor size, bp
5	164 174	164 174	167 186	167 186
5	174 180	174 180	186	186
4	169 174	169 174	172 191	172 191
4	174 180	174 180	167	167
4	174	174	167 186	167 186
4	174	174	172 182	172 182
4	164 174	164 174	167 182	167 182
4	164 180	164 180	167 186	167 186
4	174 180	174 180	167 186	167 186
4	174 180	174 180	172 181	172 181
4	174 185	174 185	167 182	167 182
4	174 185	174 185	181 186	181 186
3	174	174	167 172	167 172
3	164 174	164 174	172 182	172 182
3	164 174	164 174	182 186	182 186
3	174	174	167 181	167 181
3	164 180	164 180	182 191	182 191
3	174 180	174 180	177 182	177 182
3	174 180	174 180	181 186	181 186
3	174 180	174 180	181 191	181 191
3	174 185	174 185	177 181	177 181
3	174 190	174 190	167 181	167 181
3	164 174	164 174	181	181
3	174 184	174 184	181 190	181 190
